# Preparation of Pinoresinol and Dehydrodiconiferyl Alcohol from Eucommiae Cortex Extract by Fermentation with Traditional Mucor

**DOI:** 10.3390/molecules29132979

**Published:** 2024-06-23

**Authors:** Wenyi Jiang, Zhengyou He, Ruijiao Yao, Zhiyang Chen, Xia Zeng, Miao Zheng, Jing Wang, Jia Li, Yong Jiang

**Affiliations:** 1 School of Food and Biological Engineering, Chengdu University, Chengdu 610106, China; jiangwenyi@cdu.edu.cn (W.J.); yaoruijiao@cdu.edu.cn (R.Y.); 2 School of Pharmacy, Sichuan Industrial Institute of Antibiotics, Chengdu University, Chengdu 610106, China; chenzhiyang@cdu.edu.cn (Z.C.); zengxia@cdu.edu.cn (X.Z.); zhengmiao@cdu.edu.cn (M.Z.); wangjing@cdu.edu.cn (J.W.); lijia123@cdu.edu.cn (J.L.); jiangyong@cdu.edu.cn (Y.J.)

**Keywords:** Eucommiae Cortex, biotransformation, *Aspergillus niger*, *Actinomucor elegans*, pinoresinol, dehydrodiconiferyl alcohol

## Abstract

Eucommiae Cortex (EC) is frequently used alone or in combination with other active ingredients to treat a range of illnesses. An efficient technical instrument for changing cheap or plentiful organic chemicals into rare or costly counterparts is biotransformation. It combines EC with biotransformation techniques with the aim of producing some novel active ingredients, using different strains of bacteria that were introduced to biotransform EC in an aseptic environment. The high-quality strains were screened for identification after the fermentation broth was found using HPLC, and the primary unidentified chemicals were separated and purified in order to be structurally identified. Strain 1 was identified as *Aspergillus niger* and strain 2 as *Actinomucor elegans*; the main transformation product A was identified as dehydrodiconiferyl alcohol (DA) and B as pinoresinol (Pin). The biotransformation of EC utilizing *Aspergillus niger* and *Actinomucor elegans* is reported for the first time in this study’s conclusion, resulting in the production of Pin and DA.

## 1. Introduction

China traditionally uses Eucommiae Cortex (EC), a plant belonging to the genus Eucommiae and family Eucommiaceae, as a tonic herb. It belongs to the meridians of the liver and kidney and has the ability to settle the fetus, strengthen the muscles and bones, and tonify the liver and kidney. It is clinically used to treat liver and kidney deficiencies, lumbar and knee discomfort, muscle and bone weakness, lightheadedness, blood leaking during pregnancy, and fetal restlessness [1]. EC is frequently used alone or in combination with other active ingredients to treat a range of illnesses. Its principal constituents are phenylpropanoids, terpenoids, flavonoids, phenolic acids, steroids, and polysaccharides [2]; some of these compounds exhibit multiple biological activities in vivo or in vitro.

Biotransformation is an effective scientific tool for converting inexpensive or abundant organic compounds into more expensive or rare counterparts. Traditional Chinese medicine (TCM) and biotechnology have a lot of potential to advance economic development.

Few pertinent investigations have been done on the biotransformation of EC. Cordyceps militaris fermentation increases the amount of active compounds in EC and poplar flowers [3]. The cellulase enzyme system generated by Trichoderma reesei SCB18 was ideally utilized for the enzymatic saccharification and fermentation ethanol production processes based on the investigation of the enzymatic effect of EC gum extraction waste residue [4], as well as the isolation and identification of endophytic fungi from EC and their biotransformation of isosteviol [5]. Furthermore, in vivo metabolism is the subject of certain other literature investigations on the biotransformation of EC. The goal of the current study was to biotransform EC in order to create some novel active ingredients.

## 2. Results

### 2.1. HPLC Results

**Figure 1:**
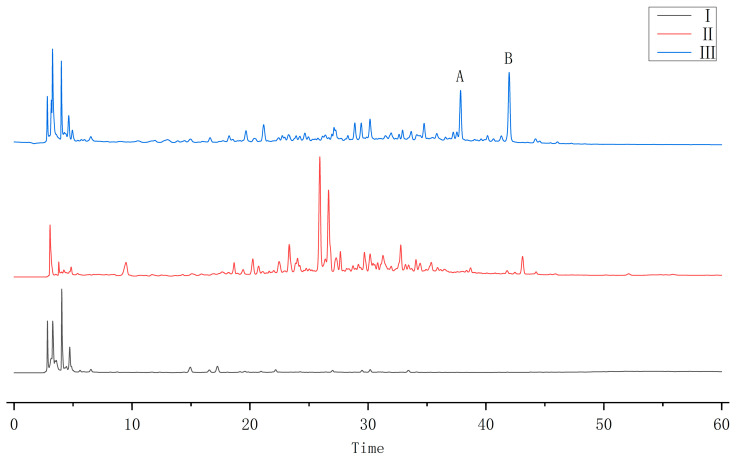
Raw chromatograms (Ⅰ is the EC-free fermentation group, Ⅱ is the EC methanol extract, Ⅲ is the fermentation group of EC methanol extracts, the fermentation strain was traditional mucor; A and B are the new peaks).

EC-free fermentation group, EC methanol extract, and fermentation group of EC methanol extracts were examined by HPLC, and the results are shown in Figure 1. The results showed that new peaks appeared in the fermentation group of EC methanol extract. Compound A and compound B, with the highest content in the fermentation broth, were selected for structural identification.

### 2.2. Strain Identification

#### 2.2.1. ITS Region Sequence

The ITS sequence is an internally transcribed spacer. In fungi, the 5.8S, 18S, and 28S rRNA genes are highly conserved, whereas ITS, being subject to less natural selection pressure, has been able to tolerate more variation during evolution and exhibits an extremely wide range of sequence polymorphisms in the vast majority of eukaryotes. At the same time, the conserved type of ITS shows relative consistency within species and more obvious differences between species, which can reflect the differences between genera and even strains. Moreover, the ITS sequence fragments are small (the lengths of ITS 1 and ITS 2 are 350 bp and 400 bp, respectively), which are easy to analyze, and have been widely used in phylogenetic analyses of different species and genera of fungi.

Sequence of strain 1 ITS region: TTCCGTAGGGTGAACCTGCGGAAGGATCATTACCGAGTGCGGGTCCTTTGGGCCCAACCTCCCATCCGTGTCTATTGTACCCTGTTGCTTCGGCGGGCCCGCCGCTTGTCGGCCGCCGGGGGGGCGCCTCTGCCCCCCGGGCCCGTGCCCGCCGGAGACCCCAACACGAACACTGTCTGAAAGCGTGCAGTCTGAGTTGATTGAATGCAATCAGTTAAAACTTTCAACAATGGATCTCTTGGTTCCGGCATCGATGAAGAACGCAGCGAAATGCGATAACTAATGTGAATTGCAGAATTCAGTGAATCATCGAGTCTTTGAACGCACATTGCGCCCCCTGGTATTCCGGGGGGCATGCCTGTCCGAGCGTCATTGCTGCCCTCAAGCCCGGCTTGTGTGTTGGGTCGCCGTCCCCCTCTCCGGGGGGACGGGCCCGAAAGGCAGCGGCGGCACCGCGTCCGATCCTCGAGCGTATGGGGCTTTGTCACATGCTCTGTAGGATTGGCCGGCGCCTGCCGACGTTTTCCAACCATTCTTTCCAGGTTGACCTCGGATCAGGTAGGGATACCCGCTGAACTTAAGCATATCAATAAGT.

Sequence of strain 2 ITS region: CTTCCGTAGGTGAACCTGCGGAAGGATCATTAAATAAAACTTGAGGGGAAACTGAGCTTACGGGCTTGGTTTTTCTCTTATTTTTTACCGTGAACTGTCTTATAGCATGGCGCTAGTAGAGATGCCTGAGCCACCATACGGGGTAGGCGGCACAGGATGATTTTAATCGAAGCCATGGTCAAGCCGACTTTTTTTCAGCTTGGTACCCCAAAAATTAATTATTCTACCAAATGAATTCAGTATTAATATTGTAACATGGGCTCGCTGAAAGGTGGCCTATAAAACAACTTTTAACAACGGATCTCTTGGTTCTCGCATCGATGAAGAACGTAGCAAAGTGCGATAACTAGTGTGAATTGCATATTCAGTGAATCATCGAGTCTTTGAACGCATCTTGCACCTGCTGGTATTCCAGCAGGTACGCCTGTTTCAGTATCAGAAACAACTCTTCCCTTAAGATTTTTTTCTTAAGGGGACATTGAGGGTATCTGGCTTAGAAGTAAAATCTCTAGCCCGGAGACGCTTTAAATGACTAAAGGCCTGCAAGCCAAAGTTTGATTGCGCCTGAACTTTTTCTTAATTTCAAGCGAAAGCTCTTGCGAACTAGAACTTTATTATTGCCTTGGGGGCCTCCCAAAGAAAACATTCAACAACTTGATCTGAAATCAGGTGGGATTACCCGCTGAACTTAAGCATATC.

#### 2.2.2. Phylogenetic Analysis of Strains

Strain 1: The inference of evolutionary relationships is done using the neighbor-joining method [6]. In the bootstrap test (1000 repetitions of the experiment), the percentage of repetitive trees in which the relevant classification units were clustered together is shown next to the branches [7]. Trees were drawn to scale, with branch lengths in the same units as the evolutionary distances used to infer phylogenetic trees. Evolutionary distances were calculated using the maximum composite likelihood method [8]; the unit is the number of base substitutions per site. The analysis involved 22 nucleotide sequences, and all sites containing missing data and gaps were excluded (complete deletion option). There were 445 loci in the final dataset. Evolutionary analyses were performed in the MEGA X software [9].

**Figure 2:**
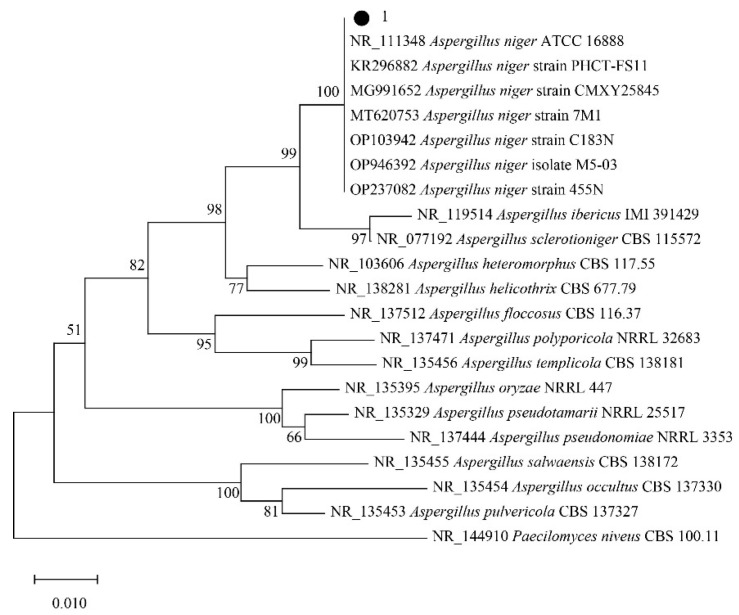
Phylogenetic analysis of strain 1.

The results are shown in Figure 2, with the black circle representing strain 1 in this study. The total branch length of the optimal tree is 0.39. Strain 1 clustered with *Aspergillus niger* of *Moniliaceae* and had 100% branching confidence. *Aspergillus ibericus* is closely related to *Aspergillus sclerotioniger* on a branch that is sister to that of *Aspergillus niger*. Other members of the *Moniliaceae* are slightly more distant from strain 1. Therefore, strain 1 is *Aspergillus niger*.

Strain 2: Analyzed as above, strain 2 involves 25 nucleotide sequences, and all loci containing missing data and gaps were excluded (complete deletion option). There were 333 loci in the final dataset. Evolutionary analyses were carried out using MEGA X software.

**Figure 3:**
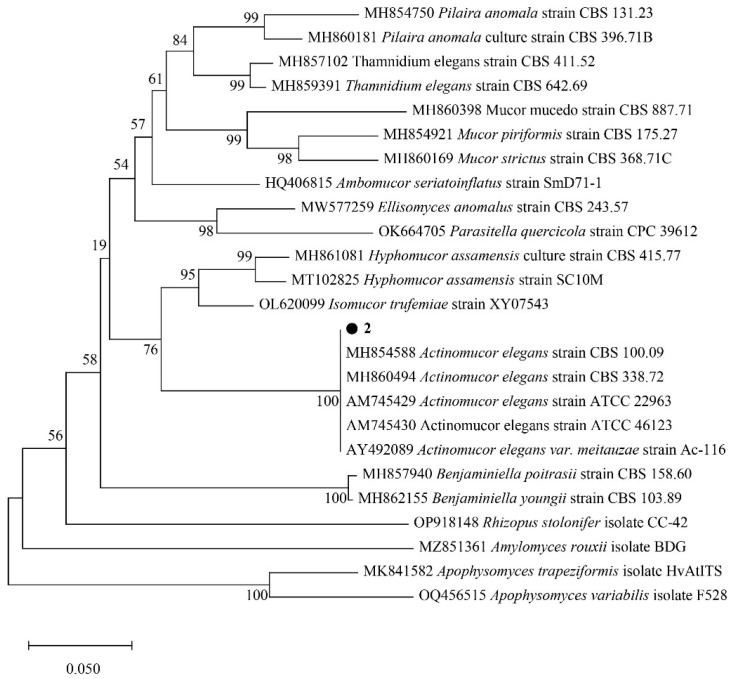
Phylogenetic analysis of strain 2.

The results are shown in Figure 3, with black circles representing strain 2 in this study. The total branch length of the optimal tree was 1.58255453. Strain 2 clustered with *Actinomucor elegans* of mucormycosis and had 100% branching confidence. *Hyphomucor* assamensis is on a branch with *Isomucor trufemiae* and is sister to the branch of *Actinomucor elegans*, which is closer in affinity. Members of other genera of mucormycosis are slightly more distant from strain 2. Therefore, strain 2 is *Actinomucor elegans*.

### 2.3. Identification of A and B

#### 2.3.1. Compound A

The ^13^C-NMR (101 MHz, MeOD) spectrum gave a total of 20 carbon signals, including 2 methyls, 9 hypomethyls, 2 methylene groups, and 7 quaternary carbons. The low-field region carbon signals δ_C_ 147.8 (C-4′), 147.7 (C-3), 146.2 (C-4), and 144.1 (C-3′) are the aromatic carbon signals of the four oxygen-linked signals; δ_C_ 130.6 (C-7′) and 126.1 (C-8′) for a pair of alkenyl carbon signals; δ_C_ 87.9 (C-7) for a hypomethyl carbon signal linked to oxygen; δ_C_ 63.5 (C-9) and 62.5 (C-9′) are two oxygen-linked methylene carbon signals; while δ_C_ 55.3 (3-OCH_3_) and 54.9 (3′-OCH_3_) are two methoxy carbon signals.

The ^1^H-NMR ((400 MHz, MeOD) spectrum showed two methoxy proton signals δ_H_ 3.89 (3H, s, 3-OCH_3_) and 3.83 (3H, s, 3′-OCH_3_); δ_H_ 5.54 (1H, d, *J* = 6.3 Hz, H-7) in the mid-field region is an oxygen hypomethyl proton signal. In addition, in the high-field region, δ_H_ 6.56 (1H, d, *J* = 15.8 Hz, H-7′) and 6.25 (1H, dt, *J* = 15.7, 5.8 Hz, H-8′) are a pair of alkenyl hydrogens proton signals, while δ_H_ 7.00–6.95 (3H, m, H-2, 2′, 6′), 6.84 (1H, d, *J* = 8.2 Hz, H-6), and 6.79 ( 1H, d, *J* = 8.1 Hz, H-5) are the five aromatic hydrogen proton signals on the benzene ring.

The substance was determined to be dehydrodiconiferyl alcohol (DA) through the synthesis of the NMR data and comparison with the existing literature [10].

The mass spectral as well as NMR data are given below: ESI-MS *m/z*: 381.13038 [M + Na]^+^; ^1^H NMR (400 MHz, MeOD) δ 7.00–6.95 (3H, m, H-2, 2′, 6′), 6.84 (1H, d, *J* = 8.2 Hz, H-6), 6.79 (1H, d, *J* = 8.1 Hz, H-5), 6.56 (1H, d, *J* = 15.8 Hz, H-7′), 6.25 (1H, dt, *J* = 15.7, 5.8 Hz, H-8′), 5.54 (1H, d, *J* = 6.3 Hz, H-7), 4.22 (2H, d, *J* = 5.7 Hz, H-9), 3.89 (3H, s, 3-OCH_3_), 3.88–3.85 (2H, m, H-9), 3.83 (3H, s, 3′-OCH_3_), 3.51 (1H, m, H-8); ^13^C NMR (101 MHz, MeOD) δ 147.8 (C-4′), 147.7 (C-3), 146.2 (C-4), 144.1 (C-3′), 133.1 (C-1), 131.2 (C-5′), 130.6 (C-7′), 128.9 (C-1′), 126.1 (C-8′), 118.3 (C-6), 115.1 (C-6′), 114.8 (C-5), 110.6 (C-2′), 109.1 (C-2), 87.9 (C-7), 63.5 (C-9), 62.5 (C-9′), 55.3 (3-OCH3), 54.9 (3′-OCH_3_), 53.8 (C-8).

**Figure 4:**
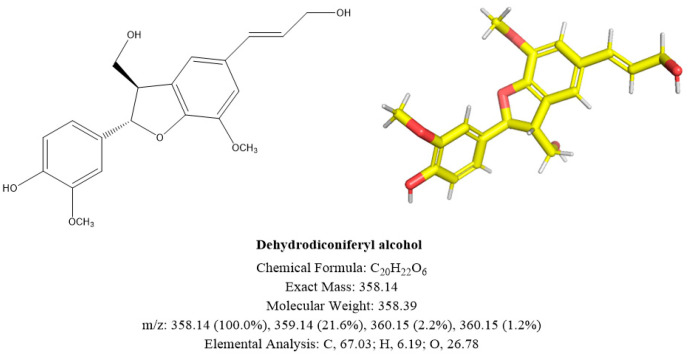
Structural diagram of **compound A**. The depicted absolute configuration was not determined experimentally and is therefore putative.

NMR and UPLC-MS results are shown in Appendix A. The structure is shown in Figure 4.

#### 2.3.2. Compound B

The ^13^C-NMR (101 MHz, MeOD) spectrum gave a total of 20 carbon signals, including 2 methyls, 10 hypomethyls, 2 methylene groups, and 6 quaternary carbons. The low-field region carbon signals δ_C_ 147.7 (C-4, 4′) and 145.9 (C-3, 3′) are the aromatic carbon signals of the linked oxygen; δ_C_ 132.4 (C-1, 1′), 118.7 (C-6, 6′), 114.7 (C-5, 5′), and 109.6 (C-2, 2′) are the rest of the aromatic carbon signals on the benzene ring, and δ_C_ 55.0 (3, 3′-OCH_3_) is the characteristic peak of the methoxy carbon signal.

The ^1^H-NMR ((400 MHz, MeOD) spectrum showed a characteristic peak of methoxy proton signal δ_H_ 3.86 (6H, s, 3, 3′-OCH_3_); δ_H_ 4.71 (2H, d, *J* = 4.0 Hz, H-7, 7′) in the mid-field region is the hypomethyl proton signal of the oxygen-linked. In addition, δ_H_ 4.23 (2H, td, *J* = 6.6, 2.4 Hz, H_a_-9, 9′) and 3.83 (2H, overlapped, H_b_-9, 9′) are the methylene proton signals for the consecutive oxygen. A set of characteristic peaks δ_H_ 6.96 (2H, d, *J* = 1.9 Hz, H-2, 2′), 6.82 (2H, dd, *J* = 8.1, 1.9 Hz, H-6, 6′) and 6.78 (2H, d, *J* = 8.1 Hz, H-5, 5′) of the ABX coupled system can be clearly seen in the high-field region, suggesting that there is a trisubstituted benzene ring structure in this structure.

The substance was determined to be pinoresinol (Pin) through the synthesis of the NMR data and comparison with the existing literature [11].

The mass spectral as well as NMR data are given below: ESI-MS *m*/*z*: 381.13038 [M + Na]^+^; ^1^H NMR (400 MHz, MeOD) δ 6.96 (2H, d, *J* = 1.9 Hz, H-2, 2′), 6.82 (2H, dd, *J* = 8.1, 1.9 Hz, H-6, 6′), 6.78 (2H, d, *J* = 8.1 Hz, H-5, 5′), 4.71 (2H, d, *J* = 4.0 Hz, H-7, 7′), 4.23 (2H, td, *J* = 6.6, 2.4 Hz, H_a_-9, 9′), 3.86 (6H, s, 3, 3′-OCH_3_), 3.86–3.80 (2H, overlapped, Hb-9, 9′), 3.20–3.08 (2H, m, H-8, 8′); 13C NMR (101 MHz, MeOD) δ 147.7 (C-4, 4′), 145.9 (C-3, 3′), 132.4 (C-1, 1′), 118.7 (C-6, 6′), 114.7 (C-5, 5′), 109.6 (C-2, 2′), 86.1 (C-7, 7′), 71.2 (C-9, 9′), 55.0 (3, 3′-OCH3), 54.0 (C-8, 8′). 

**Figure 5:**
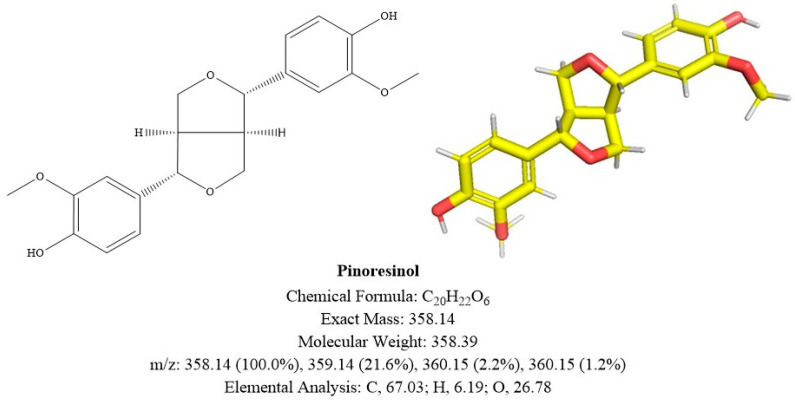
Structural diagram of **compound B**. The depicted absolute configuration was not determined experimentally and is therefore putative.

NMR and UPLC-MS results are shown in Appendix A. The structure is shown in Figure 5.

## 3. Materials and Methods

### 3.1. Materials

Chemicals, reagents, and HPLC-grade acetonitrile were purchased from Chengdu kelong chemical Co. (Chengdu, China), EC (Sichuan Zangxi tang Biotechnology Co., Sichuan, China), silica (Qingdao Wave Silica Gel Desiccant Factory, Qingdao, China), AB-8 macroporous adsorption resin (Shanghai Macklin Biochemical Technology Co., Shanghai, China), C18 (Lisure Science, Suzhou, China), methanol-D4 (Cambridge Isotope Laboratories, Inc., Tewksbury, MA, USA).

Strains: *Aspergillus niger* CICC2462; *Saccharomyces cerevisiae* OP681675.1; for strains kept in labs. Fermented bean curd fermenting bacteria (the strain came from a workshop that fermented bean curd).

### 3.2. Methods

#### 3.2.1. Extraction of EC Components

With a pulverizer, crush some dry EC. Weighing the right amount of EC, add it to anhydrous methanol and sonicate the mixture for 30 min at a material-to-liquid ratio of 1:20. After filtration and the retention of the filtrate, the residue is once more sonicated with anhydrous methanol for 30 min at a material-liquid ratio of 1:20. Combine the two filtrates and complete the evaporation of methanol.

#### 3.2.2. Fermentation Media

EC-free fermentation group: 1000 mL culture solution containing 200 g of fresh potato (purified water was boiled for 30 min and the filtrate was extracted), analytical pure glucose 20 g, analytical pure magnesium sulfate 1.5 g, potassium dihydrogen phosphate analytical pure 3 g, and VB_1_ 40 mg.

Fermentation group of EC methanol extracts: the addition of EC extract to EC-free fermentation group. EC-free fermentation group: EC (20 mL:1 g) and autoclave (121 °C, 30 min) after configuration.

#### 3.2.3. Biotransformation

Biotransformation was carried out by inoculating three strains of *Aspergillus niger*, *Saccharomyces cerevisiae*, and fermented-bean-curd-fermenting bacteria in a sterile environment, setting up three parallel trials per group. Both the EC-free fermentation group and the fermentation group of EC methanol extracts were incubated in a shaker. Samples were taken at 0, 2, 4, 6, and 8 days of incubation time and detected by HPLC.

#### 3.2.4. Collection of Transformation Products

Fermentation solution: methanol (1:2) ultrasonic extraction for 60 min, filtered on filter paper, followed by the rotary evaporation of the solvent, and dried under reduced pressure.

#### 3.2.5. HPLC Assay

- Chromatographic conditions

Column: Unisil 5-120 C18 ultra (5 μm, 4.6 × 250 mm); mobile phase: acetonitrile (A)—0.1% phosphoric acid (B); elution gradient: (0–8 min, 6%A; 8–16 min, 6%→12%A; 16–24 min, 12%→18%A; 24–32 min, 18%→24%A; 32–40 min, 24%→30%A; 40–60 min, 30%→36%A); flow: 1 mL/min; wavelength: 227 nm; column temperature: 30 °C.

- Preparation of test solutions

EC-free fermentation group (Ⅰ) and the fermentation group of EC methanol extracts (Ⅲ): Take 20 mL of fermentation broth separately. Fermentation solution: methanol (1:2) ultrasonic extraction for 60 min, filtered on filter paper, followed by the rotary evaporation of the solvent, and dried under reduced pressure. Add methanol to configure a 10 mL solution.

EC methanol extract (Ⅱ): Weigh 1 g of EC with anhydrous methanol and ultrasonic for 30 min; the material–liquid ratio was 1:20. After filtration and the retention of the filtrate, the residue was once more sonicated with anhydrous methanol for 30 min at a material–liquid ratio of 1:20. The two filtrates were mixed and concentrated to 10 mL of solution.

The fermentation broth was screened by HPLC for the production of new compounds.

#### 3.2.6. Isolation, Purification and Identification of Strains

According to the HPLC results, the best transformed strains were screened for identification. Identification process: extraction of fungal genomic DNA; PCR amplification of ITS sequences; 3730XL sequencing assay; data analysis.

#### 3.2.7. Isolation, Purification and Identification of A and B

Isolation and purification of A and B: Fermentation solution: methanol (1:2) ultrasonic extraction for 60 min, filtered on filter paper, followed by the rotary evaporation of solvent, and dried under reduced pressure. Gradient elution was performed using AB-8 macroporous adsorbent resin. Elution gradient: ethanol content (0%, 20%, 70%, 100%). In total, 70% of the eluate was collected by the rotary evaporation of the solvent and dried under reduced pressure.

Separation on silica gel columns, elution gradient: petroleum ether, petroleum ether: methylene chloride (20:1), petroleum ether: methylene chloride (15:1), petroleum ether: methylene chloride (10:1), petroleum ether: methylene chloride (2:1), petroleum ether: methylene chloride (1:1), methylene chloride: methanol (20:1). The eluate containing A and B was collected by the rotary evaporation of the solvent and dried under reduced pressure.

C18 reverse column purification, elution gradient: methanol concentration (5%, 10%, 15%, 20%, 25%, 30%, 35%, 40%). The eluate containing A and B was collected by the rotary evaporation of the solvent and dried under reduced pressure.

Structural identification of A and B: A and B were subjected to NMR and UPLC-MS structure identification.

### 3.3. Experimental Flow

**Figure 6:**
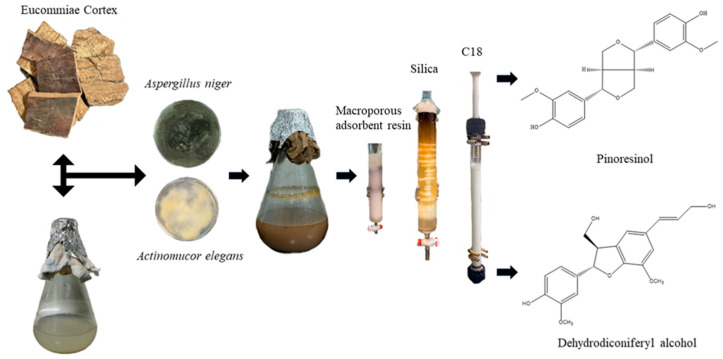
Experimental flow.

The experimental flow is shown in Figure 6.

## 4. Discussion

The Pin is a bioactive lignan widely found in many dietary plants and has been reported to have antifungal, anti-inflammatory, antioxidant, and hypoglycemic activities. Pin shows anti-tumor effects by inducing apoptosis and inhibiting invasion in HepG2 cells [12]. It improves memory deficits and promotes hippocampal LTP induction, a process that may be related to the effects of Pin on AChE and calcium inward flow production [13]. It enhances hippocampal LTP and reduces Aβ-induced LTP damage. The oral administration of Pin reduces Aβ injection-induced memory deficits, neuroinflammation, and synaptic dysfunction [14]. Pin attenuates the inflammatory response of microglia and may play a role in modulating the inflammatory state in brain diseases [15]. Pin exhibits strong anti-inflammatory properties in vitro by blocking the NF-κB signaling pathway [16]. Pin ameliorates CCl-induced acute liver injury, possibly through antioxidant activity and the inhibition of NF-κB and AP-1 down-regulation of inflammatory mediators [17].

DA acts as an agonist of the FXR and can alleviate cholestatic liver injury by activating the FXR signaling pathway, and DA has great potential for the treatment of intrahepatic cholestasis as well as a range of disorders related to lipid and glucose metabolism [18]. DA promotes BMP-2-induced osteoblast differentiation by interacting with ERα or ERβ as agonists for both receptors. DA inhibits RANKL-induced osteoclast differentiation in vitro and ovariectomy-induced bone loss in vivo, and DA also controls the osteoclast/osteoblast ratio through its estrogenic effects, which has led to its development as an effective treatment for osteoporosis [19]. DA has an inhibitory effect on VCAM-1 expression in endothelial cells through the JNK pathway, and thus, can be used as a novel drug to improve endothelial dysfunction [20]. DA ameliorates the severity of the clinical symptoms of EAE by inhibiting the infiltration of immune cells, downregulating the production of MOG-specific Th17 and Th1 cells, and suppressing the expression of various pro-inflammatory mediators in the spinal cord [21]. DA may have the potential to act as a potent antioxidant for controlling Nrf2 activity and HO-1 expression and, therefore, may be useful for development as an effective drug against a variety of inflammatory diseases [22]. DA affects the expression at the RNA level of various genes involved in adipogenesis or lipogenesis; DA also regulates the production of selective cell cycle markers that affect mitotic clonal expansion by inhibiting the DNA-binding activity of C/EBPβ. These data suggest that DA may have the potential to be developed as an agent to control fat accumulation [23].

**Figure 7:**
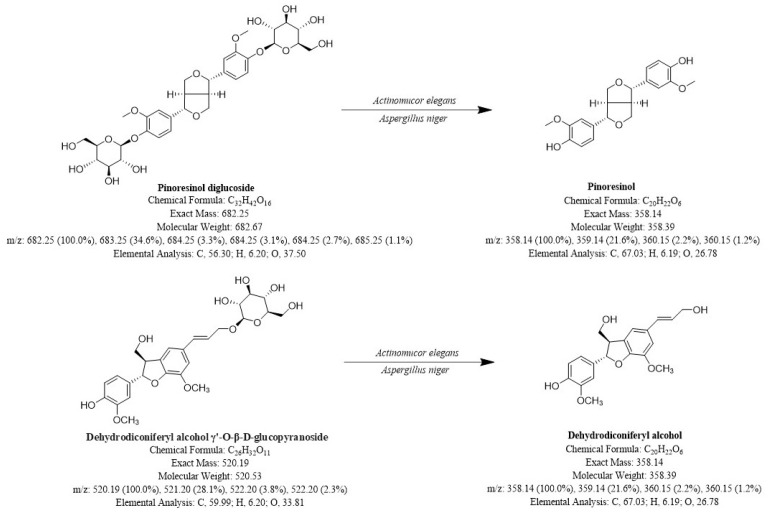
Possible generation pathways for Pin and DA.

Though there are trace elements in plants, Pin and DA have powerful anti-disease properties. This preparation method provides a reference for the mass production of Pin and DA. Figure 7 shows the potential avenues for generation.

## Figures and Tables

**Figure A1:**
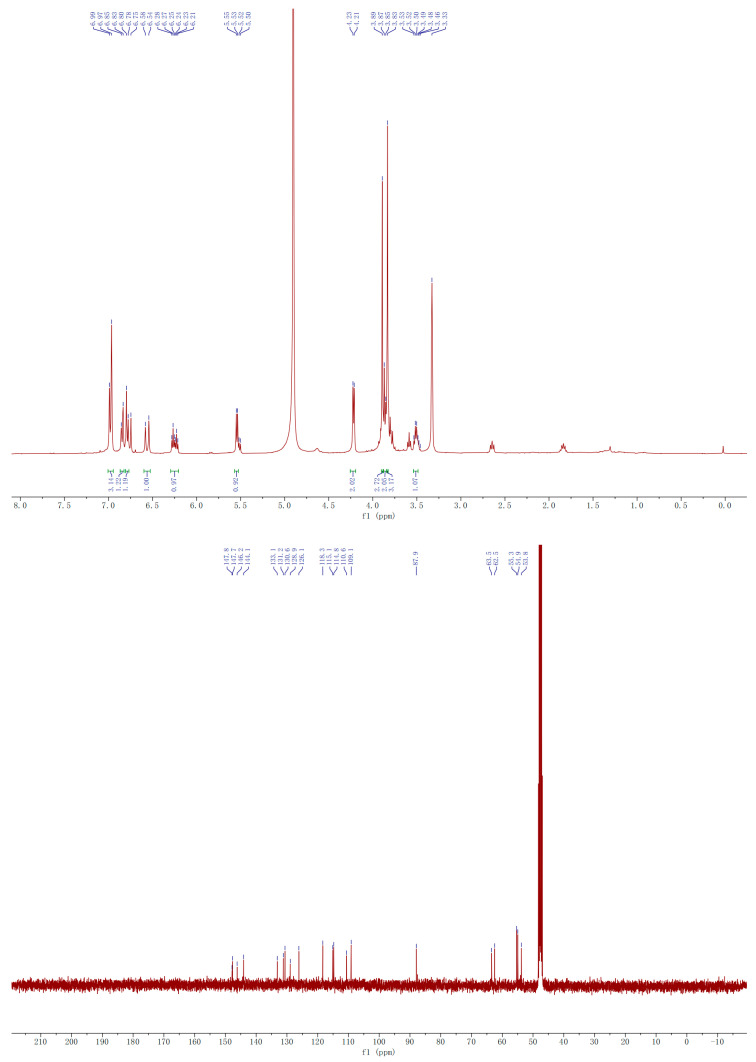
NMR of Compound A.

**Figure A2:**
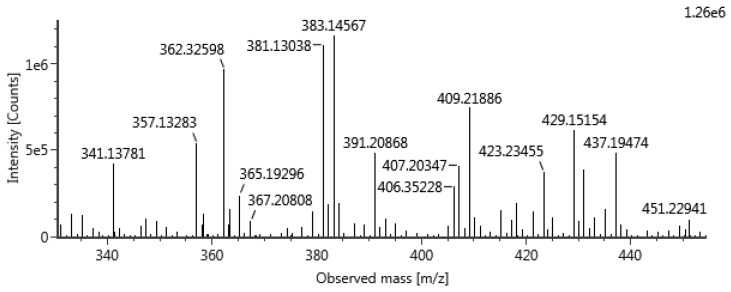
MS of Compound A.

**Figure A3:**
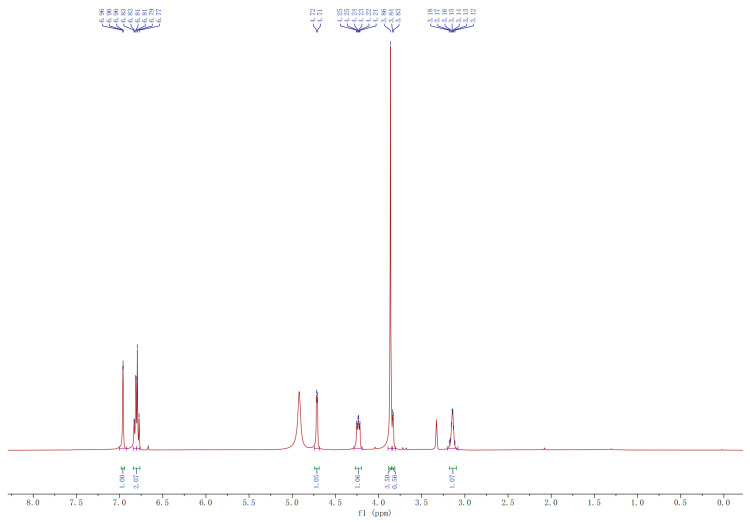

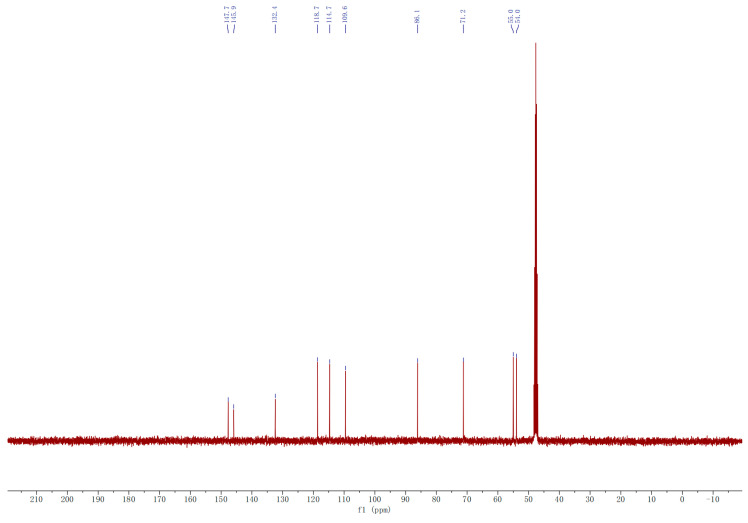
NMR of Compound B.

**Figure A4:**
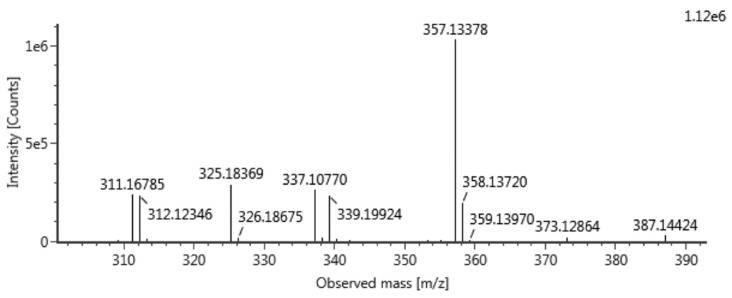
MS of Compound B.

## Data Availability

Data are contained within the article.
