# Correction: Jiang et al. Preparation of Pinoresinol and Dehydrodiconiferyl Alcohol from Eucommiae Cortex Extract by Fermentation with Traditional Mucor. *Molecules* 2024, *29*, 2979

**DOI:** 10.3390/molecules30051081

**Published:** 2025-02-27

**Authors:** Wenyi Jiang, Zhengyou He, Ruijiao Yao, Zhiyang Chen, Xia Zeng, Miao Zheng, Jing Wang, Jia Li, Yong Jiang

**Affiliations:** 1 School of Food and Biological Engineering, Chengdu University, Chengdu 610106, China; 2 School of Pharmacy, Sichuan Industrial Institute of Antibiotics, Chengdu University, Chengdu 610106, China

1. Figure Legend

In the original publication [1], there was a mistake in the legend for Figures 4 and 5. The previous description was short and not clear enough. The correct legend appears below.

**Figure 4.** Structural diagram of **compound A**. The depicted absolute configuration was not determined experimentally and is therefore putative.

**Figure 5.** Structural diagram of **compound B**. The depicted absolute configuration was not determined experimentally and is therefore putative.

2. Error in Figure

In the original publication, there was a mistake in Figures 5 and 7 as it was being published, with inaccurate description of compound names due to a previous oversight. The corrected versions of Figure 5 and Figure 7 appear below. 

**Figure 5:**
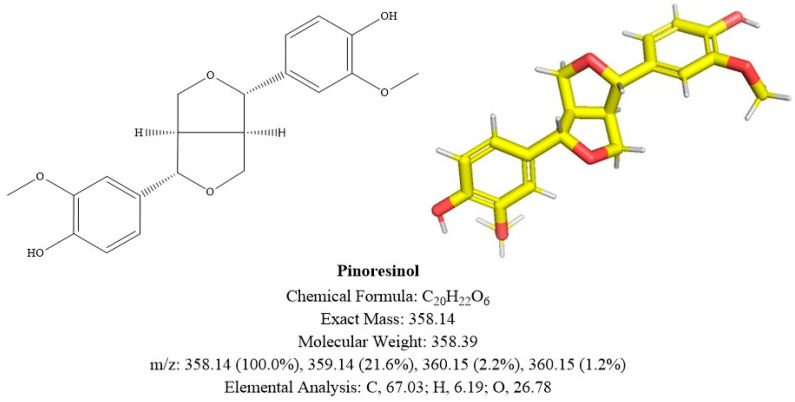
Structural diagram of **compound B**. The depicted absolute configuration was not determined experimentally and is therefore putative.

**Figure 7:**
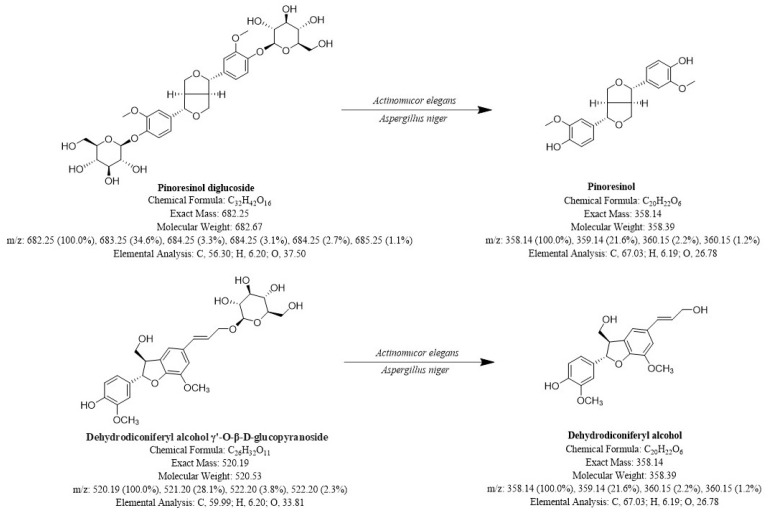
Possible generation pathways for Pin and DA.

3. Text Correction

There were four errors in the original publication. Due to an oversight on our part, the descriptions in the article were not accurate enough.

A correction has been made to Abstract:

Strain 1 was identified as *Aspergillus niger* and strain 2 as *Actinomucor elegans*; the main transformation product A was identified as dehydrodiconiferyl alcohol (DA) and B as pinoresinol (Pin).

A correction has been made to 2. Results, 2.2. Strain Identification, 2.2.2. Phylogenetic Analysis of Strains, paragraph 3 and the first sentence of paragraph 4:

Strain 2: Analyzed as above, strain 2 involves 25 nucleotide sequences, and all loci containing missing data and gaps were excluded (complete deletion option). There were 333 loci in the final dataset. Evolutionary analyses were carried out using MEGA X software.

The results are shown in Figure 3, with black circles representing strain 2 in this study.

A correction has been made to 2. Results, 2.3. Identification of A and B, 2.3.1. Compound A, paragraph 3:

The substance was determined to be dehydrodiconiferyl alcohol (DA) through the synthesis of the NMR data and comparison with the existing literature [10].

A correction has been made to 2. Results, 2.3. Identification of A and B, 2.3.2. Compound B, paragraph 3:

The substance was determined to be pinoresinol (Pin) through the synthesis of the NMR data and comparison with the existing literature [11].

4. References

Due to corrections in the content of the article, it was necessary to switch the order of the contents of references [10,11] in order to update the reference order. With this correction, the order of some references has been adjusted accordingly. 

The authors state that the scientific conclusions are unaffected. This correction was approved by the Academic Editor. The original publication has also been updated.
